# The cost of the reemergence of monkeypox: An overview of health financing in Africa

**DOI:** 10.1016/j.hpopen.2024.100132

**Published:** 2024-11-24

**Authors:** Taiwo Oluwaseun Sokunbi, Elijah Orimisan Akinbi

**Affiliations:** Young Researchers Hub, Nigeria; Obafemi Awolowo University Teaching Hospital Complex, Nigeria

Dear Editor,

## 1 Introduction

Following the global surge in monkeypox (mpox) cases in 2022, the World Health Organization (WHO) declared that mpox was no longer a public health emergency of international concern. However, the WHO emphasized the need for a sustainable, long-term approach to managing the disease [1]. In August 2024, an outbreak of mpox re-emerged, prompting the WHO to once again declare it a public health emergency of international concern [2]. These recurring outbreaks place a significant strain on healthcare financing in Africa, leading to inadequate allocation of resources within an already overburdened healthcare system and raising the costs of out-of-pocket health expenses.

The financial implications of an epidemic outbreak cannot be overstated. For example, in response to the COVID-19 pandemic, some African countries developed distinct health financing policies as part of their efforts to combat the pandemic and advance universal health coverage (UHC). While the financial impact of the pandemic has been severe worldwide, different regions and countries have experienced varying levels of impact, influenced by their specific health financing policies. Countries or regions that were less affected financially and economically by COVID-19 often share a common characteristic: robust health financing policies [3]. This study aims to provide an overview of health financing in Africa and to examine the impact of the re-emergence of mpox on health financing in the region.

## 2 The overview of mpox re-emergence in Africa

**Fig. 1:**
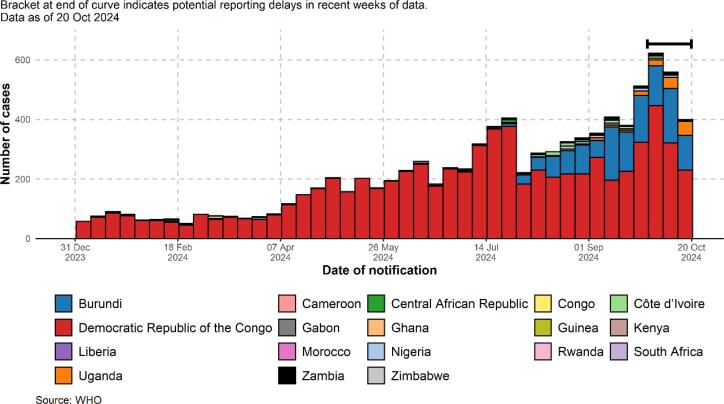
Overview of mpox re-emergence in Africa [4].

Monkeypox has re-emerged as an endemic outbreak of international concern, as declared by the WHO. Its impacts are gradually spreading across both local and global economies, affecting health system financing as a result. According to the WHO’s latest global mpox rapid risk assessment, the Democratic Republic of the Congo and its neighboring countries are categorized as high-risk, while other African nations are considered moderate-risk [1] (Fig. 1).

As of October 20, 2024, 18 countries reported a total of 9,320 confirmed mpox cases, including 34 deaths. The highest case counts in 2024 have been recorded in Burundi (1,287 cases), Uganda (153 cases), and the Democratic Republic of the Congo (7,534 cases). Due to limited diagnostic capacity in many African countries, a substantial number of suspected mpox cases that are clinically consistent with the disease go undiagnosed and unconfirmed [4]. The robustness of health financing in Africa significantly influences the economic impact of the outbreak, particularly on the people of the region.

## 3 Health financing in Africa

**Fig. 2:**
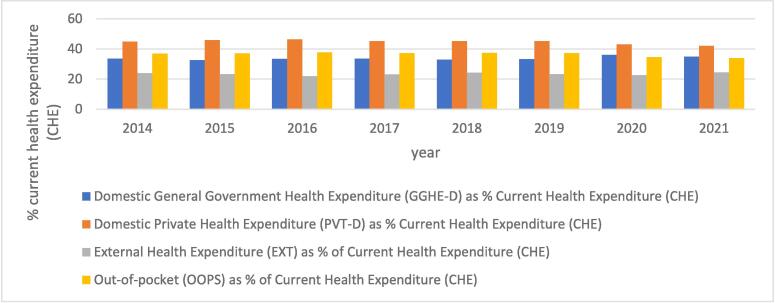
Health financing sources and corresponding expenditures over time in Africa [7].

Health financing is a crucial component of health systems, as it supports the goal of universal health coverage and enables efficient health service delivery. It influences the extent to which healthcare services are accessible to a population or country and reflects the level of financial protection provided to the population [5]. Africa bears a disproportionate share of the global disease burden, yet it is one of the regions with the lowest healthcare resource allocation. In 2015, Africa accounted for less than 1 % of total global health expenditure—ten times lower than the rest of the world [6] (Fig. 2).

Health financing sources typically include domestic governments, donors (both domestic private donors and external donors), and individuals (through out-of-pocket spending). In 2001, African governments committed to allocating at least 15 % of their annual budgets to health financing, recognizing the importance of health to economic development [5]. However, more than 20 years later, only a few countries have met this target. The average allocation of government budgets to healthcare across Africa is 7.2 %—less than half of the agreed target. In response to this limited governmental investment, other health financing sources have increased, particularly household out-of-pocket (OOP) spending. OOP expenditures make up over 35 % of current health expenditure (CHE) in Sub-Saharan Africa, the second-highest rate globally after South Asia. A 2017 report indicated that OOP spending in countries such as Nigeria, Cameroon, Equatorial Guinea, and Sudan accounted for over 70 % of CHE in Sub-Saharan Africa [5]. This reliance on OOP spending forces many households to dedicate a substantial portion of their income to healthcare, heightening their risk of poverty.

Historically, donor support has been a valuable component of health financing in Africa, yet it is unsustainable as a primary funding source. Health financing should not rely on the goodwill of other countries or organizations. In most Sub-Saharan African nations, donor funding contributes less than 20 % to CHE, though in countries like Malawi and Mozambique, donor funds account for as much as 60 % of CHE [5]. Regardless of the proportion, donor-dependent financing is particularly unreliable during health emergencies. Donor funds are often designated for specific health programs—such as vaccination, HIV/AIDS, malaria, tuberculosis, and, more recently, maternal and child health—limiting their flexibility for broader healthcare needs [5].

## 4 Impact of Mpox on health financing

Of the estimated $245 million needed to combat the growing mpox outbreak in Africa, less than 10 % has been secured [8]. Currently, no donor funds are available to alleviate the financial burden on patients [9]. In about half of African countries, over 40 % of total health expenditure comes from household out-of-pocket payments [10]. This outbreak will likely strain these payments further, as more out-of-pocket spending will be required for mpox-related hospital admissions and treatments. To control the spread of mpox, investment in vaccination, treatment, and preventive measures is essential. However, the approved vaccine is prohibitively expensive. While vaccines for many infectious diseases typically cost between $1 and $3 per dose in mass vaccination programs, the Africa Centres for Disease Control has set the price for Bavarian's mpox vaccine at $100 per dose, and the WHO has quoted it at $141 per dose [11]. This price is beyond what most African countries can afford and exceeds per capita health spending, especially considering ongoing health challenges such as cholera, measles, malaria, and HIV.

Hospitalization is also inevitable during disease outbreaks. The global case hospitalization rate for mpox following the 2022 outbreak was estimated at 14.1 %, with high variability across regions. By the outbreak period, hospitalization rates were 49.8 % before 2017, 21.7 % from 2017 to 2021, and 5.8 % during the 2022 outbreak, again with considerable variability [12]. Rising hospitalization rates will further strain already overcrowded hospitals, placing added pressure on health system infrastructure and finances. The increase in mpox-related hospitalizations adds significant financial demands, requiring more spending on inpatient care, infrastructure, workforce capacity, and preventive measures. This redirection of resources risks diverting funds from other health priorities and increasing costs for both public and private healthcare financing sources.

Africa's healthcare system, already strained by a high disease burden and limited resources, now faces an additional challenge with the rise of mpox. The continent suffers from a severe shortage of hospital beds, with many countries having fewer than 10 beds per 10,000 people [13]. This limited capacity will be further stretched as mpox cases increase, necessitating the expansion of healthcare facilities to meet the growing demand. The COVID-19 pandemic has underscored the critical need to address hospital bed shortages, particularly in Western Africa, where 8 out of 11 countries have critically low bed numbers [13]. With a limited health workforce and insufficient resources, Africa's healthcare system is ill-equipped to manage the added burden of mpox, highlighting the urgent need to increase bed availability and strengthen healthcare infrastructure.

## 5 Way Forward and Conclusion

The re-emergence of mpox in Africa presents costs that may be overwhelming for individuals and households, increasing the risk of impoverishment and making the outbreak harder to control. With inflation at record highs in many African countries [14] and rising reliance on out-of-pocket spending for mpox prevention and treatment, many households may face intensified financial strain. To address this, each African country must review its national health policy to build a sustainable and resilient financing structure that supports all citizens, particularly the most vulnerable, during health emergencies. The primary purpose of health financing policy is to shield people from severe economic impacts, especially in times of disease outbreaks [5]. African leaders should prioritize accountability by reducing dependency on donor funds to manage health emergencies. Instead, they should proactively develop and support alternative health financing mechanisms that do not place undue burden on individuals or households. A further step to demonstrate commitment is ensuring that at least 15 % of national budgets are allocated to health financing, as established in the 2001 Abuja Declaration.

**Ethical Approval**

Not applicable

**Authors’ Contributions**

T.O conceptualized the study and wrote the main manuscript.

T.O and E.O worked on the revised manuscript.

**Funding**

Not applicable.

## CRediT authorship contribution statement

**Taiwo Oluwaseun Sokunbi:** Writing – review & editing, Writing – original draft, Conceptualization, Visualization. **Elijah Orimisan Akinbi:** Writing – review & editing.

## Declaration of competing interest

The authors declare that they have no known competing financial interests or personal relationships that could have appeared to influence the work reported in this paper.
